# Rapid Analysis of Glyphosate, Glufosinate and N-Acetyl Glufosinate in Sesame by Liquid Chromatography-Tandem Mass Spectrometry

**DOI:** 10.3390/foods15122233

**Published:** 2026-06-20

**Authors:** Angela Santilio, Silvana Girolimetti

**Affiliations:** Italian National Institute of Health, Department of Environment and Health, Viale Regina Elena 299, 00166 Rome, Italy; silvana.girolimetti@iss.it

**Keywords:** highly polar pesticides, glyphosate, glufosinate, N-acetyl-glufosinate, LC/MS/MS

## Abstract

The European legislation sets the maximum residue levels for glyphosate in sesame seeds at 0.1 mg/kg (EU Regulation n. 293/2013) and for glufosinate and N-Acetyl-glufosinate expressed as glufosinate at 0.03 mg/kg (EU Regulation n. 2016/1002). The present work describes a rapid methodology to determine glyphosate, glufosinate and its metabolite and N-Acetyl-glufosinate in sesame seeds by LC/MS/MS. The method was studied in the framework of EU proficiency tests on sesame seeds. The analytical method was developed using methanol acidified with formic acid (1%, *v*/*v*) extraction with an isotope internal standard, followed by LC/MS/MS detection. The recoveries were performed in the range of 0.05–0.5 mg/kg for glyphosate and 0.02–0.2 mg/kg for glufosinate and N-Acetyl-glufosinate. All the recovery values were between 70 and 114%, which is the acceptable interval according to SANTE/11312/2021; the relative standard deviation (%RSD) values met the requirement of <20%. Linearity for each substance in solvent and matrix was studied, and the response was linear with R2 > 0.999. We considered precision, matrix effect, LOD and LOQ in the validation. All the parameters were in agreement with the acceptability criteria of the document SANTE/11312/2021. The method was considered suitable for the determination of the studied substances on sesame seeds.

## 1. Introduction

The analysis of glyphosate, glufosinate and N-acetyl glufosinate is an important issue for the laboratories responsible for the control of these substances in vegetable and animal origin products. European Union spent a lot of time studying these molecules and, to ensure safety for consumers, established maximum residue levels (MRLs) described in the Regulation (EU) n. 293/2013 [1] and Regulation (EU) n. 2016/1002 [2].

During the participation in the European proficiency test on sesame seeds, organized by the European Reference Laboratory for single-residue methods (EURL-SRMs), the laboratory studied and validated an appropriate analytical method for the determination of glyphosate, glufosinate and N-acetyl glufosinate in sesame seeds.

Sesame seeds (*Sesamum indicum* L.) are a widely used and appreciated ingredient in the world of bakeries. Sesame seeds are able to give a particular aroma to bread, to improve crunchiness and to bring health benefits even if taken in small quantities. Given this, the monitoring of pesticide residues in a commercially important crop like sesame is extremely warranted. According to the European legislation, MRLs are set for sesame seed for glyphosate at 0.1 mg/kg [1] and for glufosinate and N-acetyl glufosinate expressed as glufosinate at 0.03 mg/kg [2].

Sesame cultivation requires suitable climatic conditions for growth, such as high and constant temperatures throughout the growing cycle. For this reason, the plant is cultivated in areas such as Asia and Africa. In some intensive agricultural practices, glyphosate is used as a pre-harvest herbicide, which could lead to a risk of contamination of agricultural crops and final products, particularly in crops intended for export. Glyphosate has also been found in food products, including sesame seeds, raising concerns about consumer safety. In 2020, food safety alerts emerged regarding sesame seeds containing pesticide residues such as 2-chloroethanol and ethylene oxide, including glyphosate, which led to batch recalls, particularly in the organic sector, which must meet strict purity standards. The presence of these substances in sesame seeds from India has raised concerns within the European Union to ensure consumer safety.

The topic has interested laboratories responsible for the control of the development of analytical methods for the determination of difficult-to-analyze molecules (including glyphosate and its metabolites).

Sesame seeds are considered oily matrices, and the analysis of polar molecules such as glyphosate NAcGlufosinate is complex because it is necessary to obtain an injectable eluate free of or containing oily content that can be detected by LC/MS/MS. Available analytical methods involve extraction, purification, and derivatization steps that require lengthy analysis times and the use of significant amounts of solvents and reagents. For this reason, it is necessary to develop analytical methods capable of obtaining extracts rich in analyte and low in matrix content. The application of polar and analyte-selective solvents is a useful tool for the analysis of polar compounds such as glyphosate and its metabolites.

Glyphosate, glufosinate and its metabolite N-acetyl glufosinate due to their low molecular weight, zwitterionic nature and high polarity cannot be analyzed with multiresidue methods; therefore, the use of specific methods (single-residue methods) is required.

In this area, some authors studied specific analytical methods. Eichhorn et al. [3] studied a method applying an online SPE-LC/MS/MS method using ZrO2 (zirconia) as an adsorbent for the analyte. The methodology is suitable for the analysis of highly polar molecules such as glyphosate, AMPA and metabolites in complex matrices. In another paper, Schafer et al. [4] describe the analysis of highly polar pesticides in plant and animal origin products using the CE/MS/MS technique to improve separation between studied compounds and improve the sensitivity.

Several authors described analytical methods for the determination of glyphosate, glufosinate and its metabolites using techniques such as liquid chromatography mass spectrometry [5,6,7,8,9,10,11,12,13,14,15,16,17,18,19,20,21,22,23,24,25] or liquid chromatography with fluorescence detection [17,26,27] or liquid chromatography with UV detection after previous derivatization [28].

Most of the authors studied analytical methods in several matrices, such as vegetable (rice, maize, soybeans, oat, or lettuce), fruits (grapes, orange, tea, or coffee beans), wine, milk and beer, and plant-based milk.

Hogendoorn et al. [26] studied an analytical method in cereals. The authors analyzed cereals such as wheat, rye, barley, oat, linseed, rape seed and soybeans by derivatization with 9-fluoronylmethyl chloroformate and liquid chromatography with fluorescence detection. The method developed is useful and analyzes the compound after derivatization, extraction and cleanup steps.

Lopez et al. [15,16] studied an analytical method for the determination of several pesticides, glyphosate, glufosinate and its metabolites in fruit, vegetables and cereal (oat) and plant-based milk, wine and beer. The methods well determine the pesticide residues in several matrices, even if the use of the HILIC column required stabilization of the column with many injections. Other authors [19,22] used the HILIC column to detect pesticide residues in several matrices, and they described the problem of stability, retention time and shapes of the peaks.

A validation and application method described by Yang Liao et al. [14] studied an analytical method to detect glyphosate and glufosinate in various foods. In this method the authors used an extraction derivatization with 9-fluorenylmethylchloroformate followed by purification on solid-phase extraction; the determination was performed using isotopic-labeled analytes and LC/MS/MS. The method gives good recoveries and %RSD, but it uses dichloromethane as an extraction solvent in a mixture with water and methanol that are now not very common for monitoring laboratories, together with the derivatization.

The online solid-phase extraction coupled with liquid chromatography tandem mass spectrometry described by Eichhorn [3] gave good results in terms of validation, but it is not very useful for a monitoring laboratory that usually needs a rapid and easy method for the analysis.

During the bibliographic research, we did not find papers on sesame seeds; for this, we considered it important to study an analytical method able to determine glyphosate, glufosinate and N-acetyl glufosinate in sesame seeds. We studied a rapid and easy method able to obtain an extract useful for the determination by liquid chromatography-coupled triple quadrupole mass spectrometry (LC/MS/MS). The analysis for the analytes was performed by a rapid extraction and determination by a Raptor column. This column was also used in other applications (wheat and rice flour) for the glyphosate analysis with good performance [25]. For this reason, we used a Raptor column to detect glyphosate, glufosinate and its N-acetyl glufosinate in sesame seeds. We studied linearity, accuracy, intraday precision, matrix effect and LOQ, and all the parameters are in compliance with the acceptability criteria of the document SANTE/11312/2021 [29]. In this work no robustness was tested. The performance of the method was tested for the most significant validation parameters and the robustness of the method was not studied, leaving the relevant laboratories with a more complete evaluation for accreditation purposes. The novelty of this study was the implementation of the analytical method for the analysis of the sesame seed sample of the EUPT-SRM. The method described in this article, although it does not lead to any particular innovations for the analysis of glyphosate, glufosinate and NAcGlufosinate residues, is useful for monitoring laboratories that require rapid and easy-to-apply methods to provide immediate results. The application of the QueCHErs methodology and the LC/MS/MS technique, used by monitoring laboratories, for the analysis of target substances on sesame seeds can provide a useful contribution to routine analyses in a challenging oily matrix such as sesame seeds.

## 2. Materials and Methods

### 2.1. Chemicals and Reagents

Analytical standards from ChemService (West Chester, PA, USA) were glyphosate (purity 99.5%), glyphosate C13 (99%) N15 (purity 98%); Glufosinate (purity 99.5%), NAcd3glufosinate (purity 99.5%), N-acetyl glufosinate (purity 99.5%) and NAcd3glufosinate (purity 99.5%).

Water and acetonitrile, UPLC/MS grade, were purchased from Biosolve Chimie SARL (Dieuze, France); methanol (purity 99.9%), pesticide grade, was purchased from VWR Chemical International (Randor, PA, USA). Formic acid (purity ≥ 98%), ACS reagent, was purchased from FLUKA-Sigma Aldrich (USA) (Merck, Milan, Italy), tetrasodium ethylenediamine-tetraacetate dihydrate (EDTA) (purity > 98%) was purchased from Tokyo Chemical Industry Co (Tokyo, Japan). Anotop filter 0.22 μm (Merck).

### 2.2. Standard Solutions

Stock solutions were prepared at 1 mg/mL in water for glyphosate, glufosinate and N-acetyl glufosinate. An aliquot of each stock solution was diluted to prepare the working solution at a concentration of 10 μg/mL. Internal standard of glyphosate 13C15N at 100 μg/mL and Nacetyld3 glufosinate obtained at 100 μg/mL were diluted to obtain a solution at 10 μg/mL. All the standard solutions were stored at 4 °C, protected from light, and kept in polypropylene tubes to avoid adsorption to glass.

### 2.3. Apparatus

For sample extraction, we used a vortex mixer (IKA MS3 basic; IKA Works, Inc., Wilmington, NC, USA) and a centrifuge (ALC 4237R; Milan, Italy).

The instrumental determination was performed on a Sciex Exion LC coupled to an AB Sciex Triple Quad 4500 quadrupole system (Sciex, Framingan, MA, USA). The LC/MS/MS system consisted of an AC pump (Sciex Exion LC, Framingan, MA, USA), an AC autosampler (Sciex Exion LC, Framingan, MA, USA), an AC oven column (Sciex Exion LC, Framingan, MA, USA) and an AB Sciex triple quad 4500 mass spectrometer (Sciex Framingan, MA, USA). We performed the liquid chromatography analysis using a Raptor column (100x 2.1 mm; 2.7 μm) obtained from Restek (Milan, Italy) at 0.5 mL/min flow. The column oven temperature was set at 35 °C, the autosampler temperature at 15 °C to refrigerate the samples and an injection volume of 10 μL. The mobile phase consisted of 0.5% formic acid in ultrapure water as mobile phase [A] and formic acid 0.5% in acetonitrile as mobile phase [B] in gradient mode. The initial composition was 65% phase [B], followed by ramping to 10% over 3 min; this held for 7 min, and after 5 min the mobile phase returned to the initial composition of 65%phase B, which held for 13 min. The total run time was 30 min.

### 2.4. Sample Preparation

A total of 250 g of sesame seeds purchased from an Italian market (biological agriculture) was used for recovery tests. For recoveries, we prepared 5 g of subsamples in polytetrafluoroethylene (PTFE) centrifuge tubes 50 mL and stored them at −20 °C until analysis.

The EUPT sesame seed sample was received on dry ice, and it was stored at −20 °C until analysis. Before analysis, we prepared 10 subsamples, 5 g each, stored in polytetrafluoroethylene (PTFE) centrifuge tubes 50 mL at −20 °C until analysis.

### 2.5. Analytical Procedure

A total of 5g of sample in a 50 mL PTFE tube (±0.01 g) was added with 9 mL of cold water, followed by 100 μL of isotopically labeled glyphosate (10 μg/mL) and by 100 μL of isotopically labeled glufosinate (10 μg/mL). Afterward, we added 10 mL of methanol acidified with formic acid (1%, *v*/*v*) and an additional 100 μL of formic acid. The tube was closed and shaken by vortex mixer for 1 min. The addition of methanol acidified with formic acid allowed us to suppress the formation of a gel-like layer that can compromise the accessibility of residue from the matrix. Next, we added 1 mL of EDTA (10%, *v*/*v*) aqueous solution, and the tube was closed and shaken by vortex mixer for 3 min. We added EDTA for the complexation of metal ions, such as calcium and magnesium, which positively affects the analysis of these studied compounds. Then, the tube was centrifuged at 4000 rpm for 5 min. The organic phase was put in a 10 mL PTFE tube and frozen out at −20 °C for one night. The low temperature reduces the solubility of interfering matrix compounds, resulting in increased precipitation, and this facilitates the filtration step. After freeze-out cleanup, the organic phase was centrifuged at 4000 rpm for 5 min and 2 mL of the organic phase was separated in a 10 mL PTFE tube. In this step, to avoid the redissolution of matrix compounds, it is important to transfer 2 mL of the cold supernatant while the extract is still cold. Afterward, we added 2 mL of acetonitrile and shook by vortex mixer for 1 min and centrifuged at 4000 rpm for 5 min. The addition of acetonitrile allowed us to remove proteins and lipids, obtaining a final extract easy to filter and inject. Finally, the organic phase was filtered through an Anotop 0.22 μm filter and injected into LC/MS/MS.

Recovery tests: We prepared 5 g of sesame seed subsample in a 50 mL PTFE tube. Each sample was spiked with an appropriate quantity of standard solution to obtain fortification levels of 0.05 mg/kg, 0.02 mg/kg, 0.1 mg/kg, 0.2 mg/kg and 0.5 mg/kg for glyphosate, glufosinate and NAcglufosinate. For all spiked levels, 100 μL of isotopically labelled glyphosate and glufosinate (10 μg/mL) were added.

## 3. Results

The mass spectrometry parameters and liquid chromatography parameters were studied before proceeding with the application of the method.

For mass spectrometry, we studied, through the software Version 1.6.3 of the instrument, the parameters related to the accuracy of the main ions (precursor ions), fragmentation ions (product ions), as well as the corresponding declustering potential (DP), collision energy (CE), entrance potential (EP) and collision cell exit potential (CXP).

For each analyte, a solution at a concentration of 1 μg/mL was directly injected into the source of the MS/MS system. Each analyte was passed directly to the ionization source and, based on the automated program, the molecular ions and their energies were adjusted to obtain greater sensitivity. By the completion of the procedure, the optimized conditions were saved for each substance. The precursor ions for each compound were selected by isolation of the ion with equal or better than unit mass resolution. The use of Software Analyst (Sciex; Foster city, CA, USA) allows the identification of qualifier and quantifier ions. The quantifier ions were selected as the ions with the highest signal intensity and lowest noise. The qualifier ions were selected as the second-highest intensity ions to help confirm the compound′s identity. In addition, we consider that the ratio of qualifier to the quantifier is within the acceptability criteria of ±20% when compared to the reference standard. The identification of the target compounds was based on the retention time and the ratio of two MRM transitions.

Due to the nature of the analytes, we worked in ESI negative mode. The acquisition of the ions was done in multiple reaction monitoring (MRM) mode from 50 to 750 Da using the Analyst TF (Sciex; Foster City, CA, USA) software and the following parameters were applied: ion spray voltage of 4500 V; source temperature 600 °C; curtain gas (CUG) at 35 psi; gas 1 and 2 at 40 and 60 psi, respectively.

Table 1 shows the MRM conditions for each analyte that were used for the determination. The quantifier ions are in bold and qualifiers are reported.

For the liquid chromatography separation, we applied the chromatographic conditions reported in Santilio et al. [25] for the analysis of glyphosate in wheat and rice flour.

The use of the Raptor column (100 × 2.1 mm; 2.7 μm) obtained from Restek (Italy) at a flow of 0.5 mL/min gives good separation. The column oven temperature was 35 °C, the autosampler was maintained at 15 °C to refrigerate the samples and an injection volume of 10 μL was applied.

The Raptor column can switch between polar retention modes by modifications in mobile phase conditions. When using a high percentage of an organic mobile phase like acetonitrile, the stationary phase uses the hydrophilic interaction liquid chromatography (HILIC) retention mechanism. On the other hand, when the percentage of water increases, ion exchange is the dominant retention mode. The percentage of 0.5% formic acid is the optimum for the peak shape of the analytes. The mechanism of this column allows us to obtain a good compromise in terms of peak shape and stability of retention time.

The studied compounds were extracted from sesame seeds by adding water directly to the sample; this step leads to soaking and formation of a gel-like layer which hinders the accessibility of residue. To suppress this phenomenon, we added 10 mL of acidified methanol, 100 μL of formic acid and 1 mL of EDTA solution. During the extraction phase, the addition of EDTA helps to obtain optimal recoveries. The use of EDTA complexes Ca and Mg ions that are responsible for lower recoveries because they are competitive with the molecules under study. The phenomenon was studied and discussed by Schafer et al. [30]. Schafer et al. [30] conducted a study and discussed the phenomenon. To reduce the solubility of interfering matrix compounds, the application of low temperatures at −20 °C all night resulted in increased precipitation and facilitated the filtration step. Finally, the addition of 2 mL of acetonitrile allowed the removal of proteins and lipids, obtaining a final extract easy to filter and inject.

No further clean-up steps are required for this matrix because the freeze-out step with the addition of an isotopically labeled internal standard (IL-IS) allows the reduction in matrix effect.

We validated the analytical method for the following parameters: linearity, accuracy, intraday precision, matrix effect, limit of quantification (LoQ), and limit of detection (LoD). No robustness test was performed, leaving the relevant laboratories with a more complete evaluation for accreditation purposes.

### 3.1. Linearity

The linearity was studied for glyphosate, glufosinate and N-acetyl glufosinate in solvent and in matrix. Matrix-matched calibration solutions were prepared by adding the appropriate working solution to the blank extract to obtain final concentrations of 0.02, 0.05, 0.1, 0.2, and 0.5 mg/kg. For the preparation of the matrix-matched calibration solutions, a sesame seed sample was used. The sesame seed sample was extracted following the method described, and before adding the standard solutions, it was tested for the target compounds by LC/MS/MS. No interfering signal at the same retention time as the target compound was detected.

A calibration standard was also prepared in water with the same concentration as the matrix-matched calibration standard.

For the preparation of calibration solutions in water and in matrix, 100 μL of isotopically labeled glyphosate or glufosinate standard (10 μg/mL) was added at each level.

The correlation coefficient (R2), intercept and slope of the regression line were evaluated for each calibration curve.

For each calibration curve, we repeated the injection of each point three times and averaged the area values with respect to the concentration. Table 2 shows the linearity curve in medium acetonitrile and extract of sesame seeds. As shown in Table 2, good linearity was obtained for all the compounds for both solvent and matrix-matched conditions. The correlation coefficient is more than 0.999 for all the compounds for both solvent and matrix-matched conditions.

For the matrix-matched curves, we calculated the maximum residual value. For glyphosate, it is 14%, while for glufosinate it is 16%. Finally, for N-acetyl glufosinate the maximum residual value is 18%. The values are according to the acceptability criteria of maximum residual value less than 20% as described by SANTE/11312/2021 [29] document.

For the studied compounds, the slope values of the solvent calibration curves are 10 times larger than those obtained with the matrix calibration curves. Both calibration curves were prepared by adding the deuterated internal standard, which is intended to correct for ion suppression effects. It is observed that the matrix plays an important role in the slope of the curves and in the correlation between response and concentration. Consequently, the use of matrix calibration curves and isotope addition as an internal standard helps optimize the signal, obtaining robust results in quantifying such a complex matrix.

### 3.2. Accuracy and Precision

The accuracy and precision were determined by recovery assays. Fortification levels of 0.05 mg/kg, 0.1 mg/kg and 0.5 mg/kg were studied to evaluate the recoveries for glyphosate. For glufosinate and N-acetyl glufosinate the fortification levels of 0.02 mg/kg, 0.1 mg/kg and 0.2 mg/kg were studied. The fortification levels were designed to cover the LOQ, 10LOQ and an extra point. For each level, we analyzed five replicates according to the method described. The precision in the case of repeatability (relative standard deviation repeatability (RSDr) was determined by analyzing five replicate samples at each fortified level, on the same day with the same operator.

According to the document SANTE/11312/2021 [29], the acceptability criteria are recovery values ranging from 70 to 120%, and the relative standard deviation (%RSD) values <20%.

**Figure 1:**
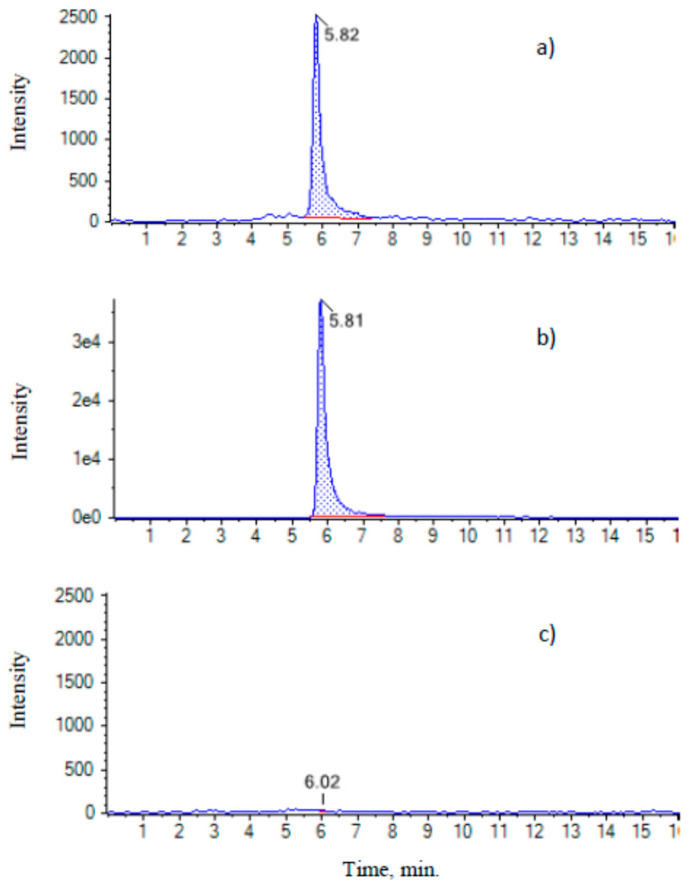
MRM chromatogram of glyphosate at 167.8 > 63.0 transition: (**a**) Glyphosate recovery at spiked level 0.5 mg/kg. (**b**) Glyphosate matrix matched standard 0.5 mg/kg. (**c**) Blank matrix (sesame seeds).

**Figure 2:**
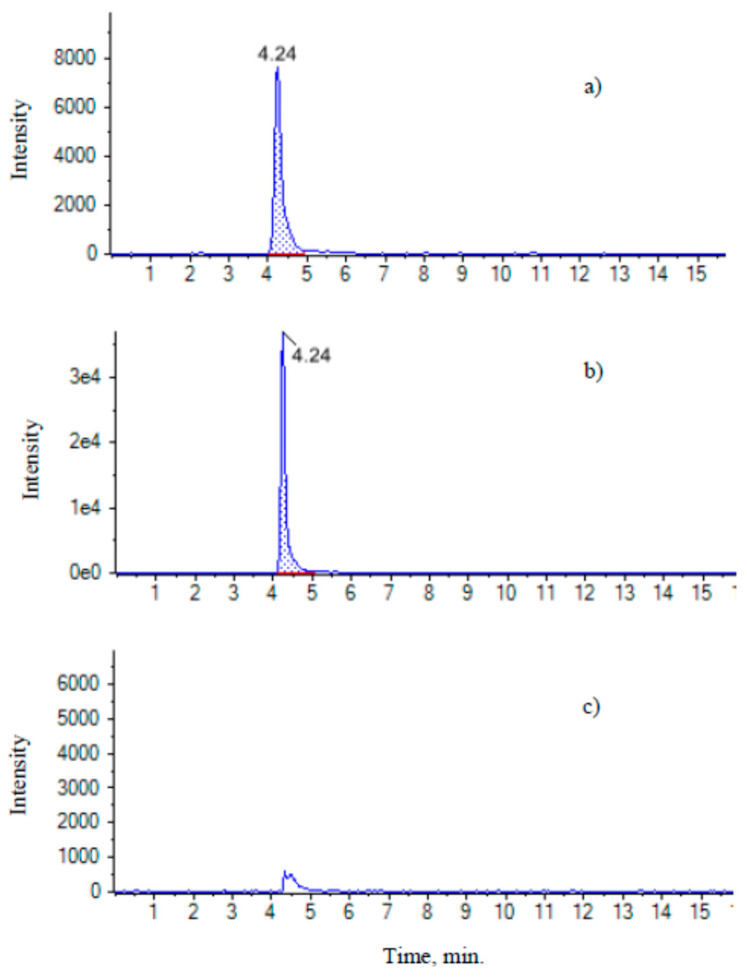
MRM chromatogram of glufosinate at 180 > 63.0 transition: (**a**) Glufosinate recovery at spiked level 0.2 mg/kg. (**b**) Glufosinate matrix-matched standard 0.2 mg/kg. (**c**) Blank matrix (sesame seeds).

**Figure 3:**
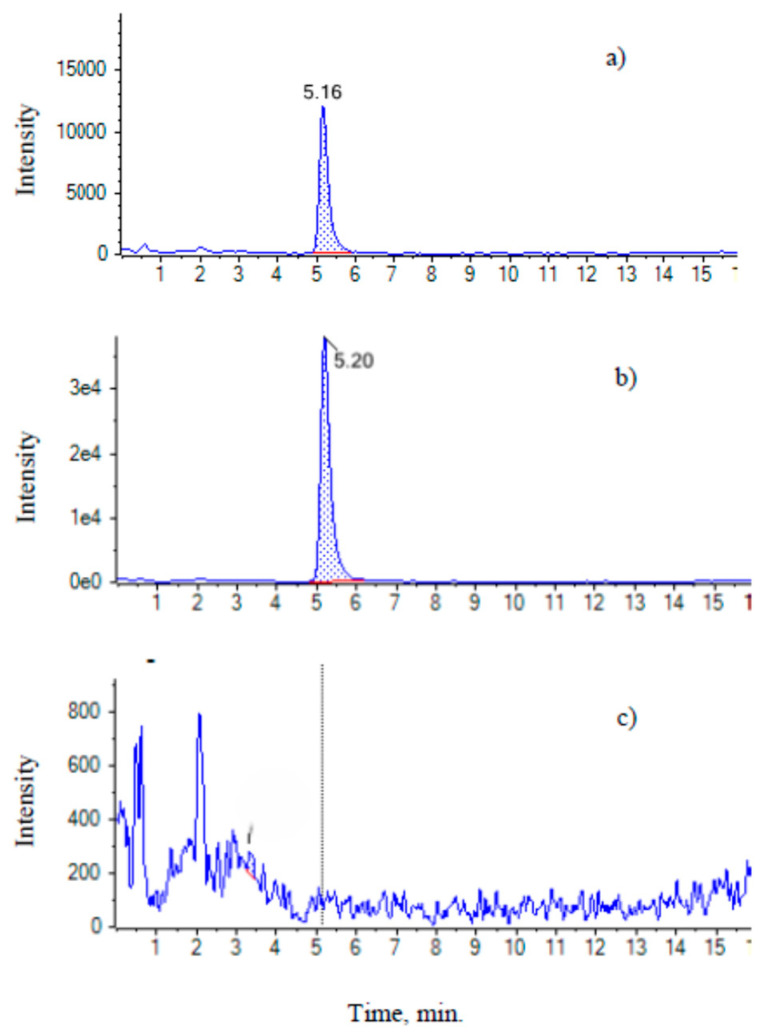
MRM chromatogram of N-acetyl glufosinate at 222.0 > 136.0 transition: (**a**) N-acetyl glufosinate recovery at spiked level 0.2 mg/kg. (**b**) N-acetyl glufosinate matrix-matched standard 0.2 mg/kg. (**c**) Blank matrix (sesame seeds).

The sesame seeds sample used for the fortification was firstly analyzed to verify the absence/presence of interfering substances at the same retention time of the studied compounds. No evidence of interfering substances was observed at the same retention times of the compounds using the quantification transitions. The quantification of the target compounds was performed using a matrix -matched external calibration solution at corresponding spiked levels. Figure 1, Figure 2 and Figure 3 also show the blank matrix for each compound.

For glyphosate good recoveries were obtained, ranging between 95 and 114%, for the fortification levels of 0.05 mg/kg, 0.1 mg/kg and 0.5 mg/kg. The relative standard deviation (RSD %) is between 4 and 7%.

For glufosinate, the recoveries were achieved between 98 and 109% within the fortification levels of 0.02 mg/kg, 0.1 mg/kg and 0.2 mg/kg. Good RSD% was obtained between 2 and 4%.

Finally, for N-acetyl glufosinate, the recoveries were obtained between 96 and 100% in the fortification range 0.02–0.2 mg/kg. Good RSD% was obtained between 1 and 3%.

The results show good agreement with the established acceptability criteria. Table 3 shows the recoveries, standard deviation and relative standard deviation for each fortification level. According to the definition of the SANTE 11312/2021 [29] document, the precision as repeatability (%RSD) was studied; we performed the tests using the same method on the same sample in a single laboratory. The results obtained are in good agreement with the information from other authors in a comparable matrix, such as soybean.

Table 4 shows the results (recoveries and reproducibility) with other literature methods.

For glyphosate in soybeans, Botero obtained a recovery of 96% with an RSD% of 5%, at a spiked level of 2.0 mg/kg, using LC/MS/MS. The value is comparable with our recovery value even if obtained at higher spiked levels.

Schafer, for glyphosate, obtained a recovery value of 87% with an RSD% = 22% at a 0.05 mg/kg spiked level. The values are different from our value, with the RSD% higher than ours at the same spiked levels, probably due to the different technique used, CE/MS/MS against LC/MS/MS.

Finally, the data from EURL-SRM shows values better than ours in terms of recovery and RSD% at the same spiked level of 0.1 mg/kg.

For glufosinate in soybeans, Schafer submitted no data, while the data from EURL-SRM show recovery and RSD% at the spiked level of 0.06 mg/kg comparable with ours at 0.1 mg/kg.

For the last compound, N-Acetyl glufosinate, Schafer obtained recovery of 86% with RSD % = 16 at the spiked level of 0.02 mg/kg. These values are quite different from ours, considering the different analytical technique used.

The data from EURL-SRM show data comparable with ours, even if a value of RSD% = 11.9 was obtained against our value of RDS% = 3.2% with the same analytical technique and spiked level.

In summary, the comparison of recoveries and RSD% with other authors shows the influence of analytical technique and matrices used.

Figure 1, Figure 2 and Figure 3 show examples of the recovery chromatograms with the matrix-matched standard solution.

Figure 1 shows the MRM chromatogram of glyphosate for recovery at a spiked level of 0.5 mg/kg (Figure 1a) for matrix-matched standard (Figure 1b) and for blank matrix (Figure 1c).

Figure 2 shows the MRM chromatogram of glufosinate for recovery at the spiked level of 0.2 mg/kg (Figure 2a), for the matrix-matched standard (Figure 2b) and for the blank matrix (Figure 2c).

Figure 3 shows the MRM chromatogram for N-acetyl glufosinate for recovery at spiked levels of 0.2 mg/kg (Figure 3a) and for the matrix-matched standard (Figure 3b) and for the blank matrix (Figure 3c).

### 3.3. Limit of Detection and Quantification

The limit of detection (LoD) was studied for all the analytes, according to the EURACHEM guidelines [32]. The LoD was calculated as the analyte concentration corresponding to three times the standard deviation. Ten independent sample blanks were fortified at 0.02 mg/kg for glufosinate and N-acetyl glufosinate, at 0.05 mg/kg for glyphosate and injected in LC/MS/MS.

The limit of quantification (LoQ) was determined as the lowest fortification level for the analyte for which acceptable accuracy (mean recoveries in the range 70–120%) and precision (RSDr ≤ 20%) could be achieved, according to the European Commission (EC) document SANTE/113112/2021 [29].

The LoD values are 0.005 mg/kg for glyphosate and 0.002 mg/kg for glyfosinate and N-acetyl glufosinate. The LoQ values are 0.05 mg/kg for glyphosate and 0.02 mg/kg for glufosinate and N-acetyl glufosinate. The limits of quantification (LoQs) are sensitive enough to meet the requirement imposed by EU Regulation for the MRLs of glyphosate and glufosinate in sesame seed.

### 3.4. Matrix Effect

Matrix effect was calculated by comparing the slope of the calibration curves in matrix extract and in solvent. Table 2 shows the slope of the calibration curves in solvent and in matrix.

The ratio between the slope from the matrix-matched calibration curve and the slope from solvent calibration was calculated according to Equation (1).

(1) $$\underline{Matrix\;matched\;calibration\;slope}\;Solvent\;calibration\;slope$$

When the ratio % is less than 20%, no matrix effect is observed; when the ratio % is between 20 and 50%, we have a medium matrix effect, and if the ratio is more than 50%, we observe a strong matrix effect.

In our case, the percentage of the signal suppression-enhancement is equal to 10.5% for glyphosate, 9.9% for glufosinate and 10.1% for N-acetyl glufosinate. The data demonstrate that no matrix effect occurred, and the use of isotope processing standard contributes to the reduction in the matrix effect [20]. As described by Kruve A [20] the matrix effect was found to be dependent on sample particle size taken from extraction. If samples are ground to very small particles, severe ionization suppression occurs. For the sesame seeds sample was homogeneous and with very small particles.

In such a complex matrix as sesame seeds (oleaginous), the matrix effect is remarkable, as we can see from the slopes of the calibration curves in solvent and matrix; consequently, it is important for quantification to use the internal isotope standard of the target compounds and the matrix standard to obtain robust results.

## 4. Discussion

The analytical method studied was very useful and versatile for the analysis of glyphosate, glufosinate and its metabolite N-acetyl glufosinate in sesame seeds using the LC/MS/MS technique. The use of the Raptor column yielded rapid analysis of glyphosate, glufosinate and NAcG, which is simple and applicable to routine analysis in monitoring laboratories. The method was tested exclusively for sesame seeds, and to evaluate the performance of the method for other sesame matrices, further investigations are needed.

Most methods in the literature determine glyphosate, glufosinate, and NAcglufosinate in matrices such as soybeans, but sesame seeds are not a common matrix, although they are of interest for monitoring. The proposed methodology allows for rapid and easy determination of target compounds by a versatile stationary phase, with acceptable results compared to the method performance.

The proposed method allows for rapid results and acceptable performance for monitoring laboratories. The performance of the method was tested for the most significant validation parameters and the robustness of the method was not studied, leaving the relevant laboratories with a more complete evaluation for accreditation purposes.

The application of the QuEChERS methodology and the LC/MS/MS technique, used by monitoring laboratories, for the analysis of target substances on sesame seeds can provide a useful contribution to routine analyses in a challenging oily matrix such as sesame seeds.

Finally, the proposed method highlights the importance of quantifying with matrix standards and with the target compound isotope, since both factors help to obtain robust quantitative results.

The method applied to the EU SRM PT on sesame seeds sample resulted in good performance and was in line with the QuEChERS methodology applied to other oil seeds. The sample contained glyphosate and glufosinate; applying the described method, the z-score values were acceptable. According to the acceptability criteria of z-score ≤ 2, for glufosinate the z-score value was 0.21 and for glyphosate was 0.46.

## Figures and Tables

**Table 1:** MRM conditions for MS/MS analysis.

Analyte	RT (min)	Precursor Ion *m*/*z*	Product Ion *m*/*z*	DP	EP	CE	CXP
Glyphosate 1	5.80	167.8	150.0	−40.0	−8.0	−14.0	−10.0
Glyphosate 2	5.80	167.8	124.0	−40.0	−8.0	−16.0	−3.0
Glyphosate 3	5.80	167.8	81.0	−40.0	−8.0	−20.0	−7.0
Glyphosate 4	**5.80**	**167.8**	**63.0**	−40.0	−8.0	−30.0	−5.0
Glyphosate isotope 1	**5.82**	**169.9**	**63.0**	−42.0	−10.0	−30.0	−6.0
Glyphosate isotope 2	5.82	169.9	126.0	−42.0	−10.0	−18.0	−3.0
Glufosinate 1	4.24	180	136	−60	−7.0	−22.0	−12.0
Glufosinate 2	4.24	180	95	−60	−7.0	−21.0	−8.0
Glufosinate 3	4.24	180	85	−60	−7.0	−23.0	−7.0
Glufosinate 4	**4.24**	**180**	**63.0**	−60	−7.0	−53.0	−5.0
N-acetyl glufosinate 1	5.18	222.0	136	−63.0	−9.0	−30.0	−13.0
N-acetyl glufosinate 2	**5.18**	**222.0**	**63**	−63.0	−9.0	−81.0	−16.0
N-acetyl glufosinate 3	5.18	222.0	59	−63.0	−9.0	−19.0	−5.0
NAceD3Glufosinate 1	5.20	225.0	137.0	−63.0	−9.0	−30.0	−13.0
NAceD3Glufosinate 2	**5.20**	**225.0**	**63.0**	−63.0	−9.0	−80.0	−16.0

**Table 2:** Linearity (solvent and matrix), regression curves (slope and intercept), correlation coefficient (R^2^) and percent residual values.

Compound	Linearity Range (mg/kg)	Medium	Regression Curve		R^2^	Residual %
			Slope	Intercept		
Glyphosate	0.05–0.5	Acetonitrile	13.086	0.0001	0.9995	--
Glyphosate	0.05–0.5	Sesame seed	1.3696	0.0187	0.9992	14
N-acetyl glufosinate	0.02–0.2	Acetonitrile	15.087	−0.0023	0.9998	--
N-acetyl glufosinate	0.02–0.2	Sesame seed	1.5227	−0.0064	0.9995	18
Glufosinate	0.02–0.2	Acetonitrile	9.6485	−0.0015	0.9999	--
Glufosinate	0.02–0.2	Sesame seed	0.9565	−0.0146	0.9988	16

**Table 3:** Average recoveries (%), standard deviation (SD) and relative standard deviation (RSD) at different spiked levels.

Crops	Spiked Levels (mg/kg)	Average Recovery (%) (n = 5)	Standard Deviation (SD)	Relative Standard Deviation (RSD%)
Glyphosate	0.05	114.2	7.7	6.7
	0.1	99.6	6.4	6.4
	0.5	95.3	4.2	4.4
Glufosinate	0.02	108.7	3.1	2.8
	0.1	87.9	3.3	3.8
	0.2	97.5	1.9	1.9
N-acetyl glufosinate	0.02	100.0	3.0	3.0
	0.1	98.9	3.2	3.2
	0.2	96.1	1.1	1.1

**Table 4:** Comparison of recoveries and reproducibility with other methods.

Matrix	Techniques	Compound	Fortification Level (mg/kg)	Recoveries (%)	RSD%
Soya bean *	LC/MS/MS	Glyphosate	2.0	96	5
Soya bean **	CE/MS/MS	Glyphosate	0.05	87	22
Soya bean **	CE/MS/MS	Glufosinate	0.06	n.d.	--
Soya bean **	CE/MS/MS	NAceGlufosinate	0.02	86	16
Soya bean ***	LC/MS/MS	Glyphosate	0.1 mg/kg	103	2
Soya bean ***	LC/MS/MS	Glufosinate	0.06 mg/kg	102	4.8
Soya beans ***	LC/MS/MS	NAce-Glufosinate	0.08	96	11.9

* Botero et al. [10]. ** Schafer et al. [4] *** EURL-SRM [31].

## Data Availability

The original contributions presented in this study are included in the article. Further inquiries can be directed to the corresponding author.
